# Severe Inflammatory Response in Myelodysplastic Syndrome and Trisomy 8 Following 23-Valent Polysaccharide Pneumococcal Vaccine Administration

**DOI:** 10.4274/tjh.galenos.2021.2020.0664

**Published:** 2021-02-25

**Authors:** Hirohisa Fujikawa, Yusuke Miyazato, Kenichiro Ebisuda, Minoru Saito

**Affiliations:** 1 The University of Tokyo, International Research Center for Medical Education, Graduate School of Medicine, Department of Medical Education Studies, Tokyo, Japan; 2 Suwa Central Hospital, Department of Internal Medicine, Tamagawa, Japan; 3 National Center for Global Health and Medicine, Disease Control and Prevention Center, Tokyo, Japan

**Keywords:** Myelodysplastic syndrome, Trisomy 8, Behçet’s disease, 23-Valent polysaccharide pneumococcal vaccine, Severe inflammatory syndrome, Severe inflammatory reaction

## To the Editor,

*Streptococcus pneumoniae* can cause serious diseases such as pneumonia, sepsis, and meningitis. Invasive pneumococcal disease (IPD) is an infection that is confirmed by the isolation of *Streptococcus pneumoniae* from a normally sterile site (e.g., blood or cerebrospinal fluid). IPD causes significant morbidity and mortality, especially in the elderly and immunocompromised patients. As a widely used vaccine worldwide, the 23-valent polysaccharide pneumococcal vaccine (PPSV23) is safe and effective for vaccination against IPD [1]. Here we report an uncommon case of severe inflammatory response in a patient with myelodysplastic syndrome (MDS) and trisomy 8 following PPSV23 vaccination.

**Figure 1:**
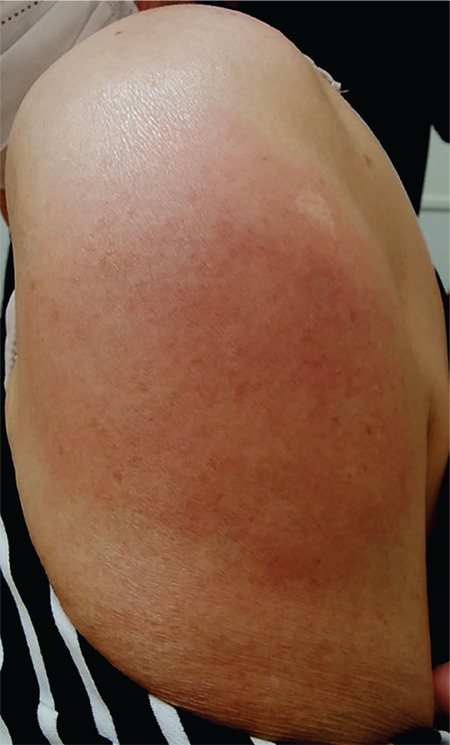
Pain, redness, and swelling at the injection site on the left upper arm.

A 65-year-old woman with MDS and trisomy 8 was admitted to the hospital because of a 2-day history of fever (39 °C) and chills. She was given her first injection of PPSV23 (Pneumovax NP^®^) about 10 h prior to the onset of symptoms. She had never received a *Streptococcus pneumoniae* polysaccharide vaccine before. On examination, she was febrile and had pain, redness, and swelling at the injection site on the left upper arm (Figure 1). Laboratory tests showed a white cell count of 14.5x10^9^/L and C-reactive protein level of 165.4 mg/L. We made the diagnosis of severe inflammatory response following PPSV23 vaccination. She was treated by cooling the affected area and administrating antibiotics, and she recovered within a few days of admission. This adverse event was reported to the Pharmaceuticals and Medical Devices Agency.

MDS is a clonal disorder of myeloid stem cells and is characterized by ineffective and dysplastic hematopoiesis that causes blood cytopenias. Although MDS is a hematological disease, it is frequently associated with various autoimmune disorders, including polymyalgia rheumatica, rheumatoid arthritis, systemic lupus erythematosus, and systemic vasculitis [2,3].

Some case studies indicate a relationship between MDS and Behçet’s disease (BD). Trisomy 8 plays a significant role in these disorders. As many as 73.7% of patients with MDS and BD had trisomy 8 [4], whereas trisomy 8 represented only 5% of all MDS cases in another report [5]. BD (or BD-like syndrome) with trisomy-8-positive MDS often forms a distinct subset of patients. Gastrointestinal lesions are very common. Contrastingly, ocular involvement and HLA-B51 positivity are relatively rare [6]. Although an etiological link between BD, MDS, and trisomy 8 has not yet been revealed, the presence of trisomy 8 seems to result in an overexpression of several cytokine genes, which enhances neutrophil function [6].

A search of the relevant literature revealed some patients with BD or suspected BD who developed severe inflammation after receiving PPSV23 (Table 1). In 2012, Hügle et al., [7] first reported four patients with BD who developed severe local and systemic inflammatory reactions to the first administration of PPSV23. No systemic adverse events were reported in their patients, who were inoculated with PPSV23 for autoimmune diseases other than BD. Although the precise pathophysiology was unclear, the authors proposed that the severe adverse reactions might have resulted from an aberrant activation of the inflammasome. To the best of our knowledge, this report is the first to describe a severe inflammatory reaction after PPSV23 vaccination in a patient with MDS and trisomy 8.

To conclude, we have described here a rare case of severe inflammatory response in a patient with MDS and trisomy 8 following the first administration of PPSV23. The present case may suggest a new BD-like presentation of trisomy-8-positive MDS. Additionally, the safety of PPSV23 for patients with trisomy-8-positive MDS should be further evaluated.

## Figures and Tables

**Table 1:**
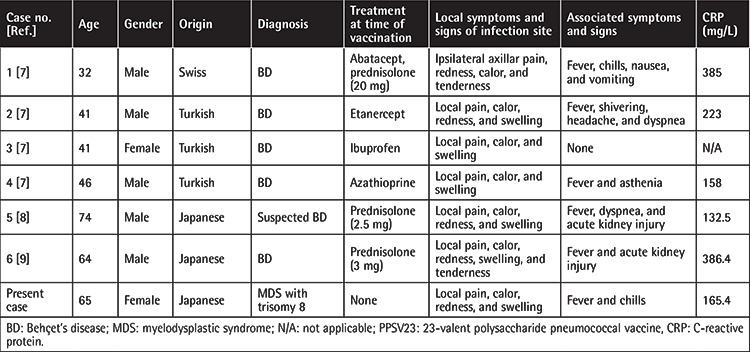
Clinical characteristics of patients with BD, suspected BD, or trisomy-8-positive MDS who developed severe inflammatory response following PPSV23 vaccination.
